# Antioxidant Treatment Induces Hyperactivation of the HPA Axis by Upregulating ACTH Receptor in the Adrenal and Downregulating Glucocorticoid Receptors in the Pituitary

**DOI:** 10.1155/2017/4156361

**Published:** 2017-05-09

**Authors:** Jessika P. Prevatto, Rafael C. Torres, Bruno L. Diaz, Patrícia M. R. e Silva, Marco A. Martins, Vinicius F. Carvalho

**Affiliations:** ^1^Laboratório de Inflamação, Instituto Oswaldo Cruz, Fundação Oswaldo Cruz, Av. Brasil, No. 4365, Manguinho, 21045-900 Rio de Janeiro, Brazil; ^2^Laboratório de Inflamação, Instituto de Biofísica Carlos Chagas Filho, Universidade Federal do Rio de Janeiro, Av. Carlos Chagas Filho, No. 373, Cidade Universitária-Ilha do Fundão, 21941-902 Rio de Janeiro, Brazil; ^3^National Institute of Science and Technology on Neuroimmunomodulation (INCT-NIM), Rio de Janeiro, Brazil

## Abstract

Glucocorticoid (GC) production is physiologically regulated through a negative feedback loop mediated by the GC, which appear disrupted in several pathological conditions. The inability to perform negative feedback of the hypothalamus-pituitary-adrenal (HPA) axis in several diseases is associated with an overproduction of reactive oxygen species (ROS); however, nothing is known about the effects of ROS on the functionality of the HPA axis during homeostasis. This study analyzed the putative impact of antioxidants on the HPA axis activity and GC-mediated negative feedback upon the HPA cascade. Male Wistar rats were orally treated with N-acetylcysteine (NAC) or vitamin E for 18 consecutive days. NAC-treated rats were then subjected to a daily treatment with dexamethasone, which covered the last 5 days of the antioxidant therapy. We found that NAC and vitamin E induced an increase in plasma corticosterone levels. NAC intensified MC2R and StAR expressions in the adrenal and reduced GR and MR expressions in the pituitary. NAC also prevented the dexamethasone-induced reduction in plasma corticosterone levels. Furthermore, NAC decreased HO-1 and Nrf2 expression in the pituitary. These findings show that antioxidants induce hyperactivity of the HPA axis via upregulation of MC2R expression in the adrenal and downregulation of GR and MR in the pituitary.

## 1. Introduction

Reactive oxygen species (ROS) are ions or small molecules containing oxygen and an unpaired electron, and this free electron confers high reactivity to oxygen. ROS production in mammals is due to the activity of endogenous pro-oxidant enzymes NADPH oxidase, xanthine oxidase, peroxisomes, and cytochrome P-450. Their product is counterbalanced by endogenous antioxidant enzymes including superoxide dismutase, catalase, glutathione peroxidase, reduced glutathione, and heme oxygenase- (HO-) 1 [1]. These antioxidant defense systems are directly regulated by nuclear factor erythroid 2-related factor 2 (Nrf2). Besides inducing the transcription of endogenous antioxidant enzymes, Nrf2 affects the homeostasis of ROS and reactive nitrogen species (RNS) through regeneration of oxidized cofactors and proteins; synthesis of reducing factors, as GSH and NADPH; and increasing redox transport, including cysteine/glutamate transport by xCT [2]. Redox imbalance is induced by disequilibrium between the production and suppression of ROS. Furthermore, excess of oxidative damage can be controlled by exogenous antioxidants such as vitamins C and E, polyphenols, carotenes, flavonoids, omega-3, and N-acetylcysteine (NAC) [3–5]. These exogenous antioxidants decrease the oxidative damage through distinct mechanisms of action. For instance, vitamin E, a nonenzymatic antioxidant, promotes a lipid peroxyl radical scavenger and maintains the integrity of long-chain polyunsaturated fatty acids in the membrane of cells [6, 7], while NAC, which is an acetylated cysteine residue, stimulates glutathione synthesis and performs as a scavenger of oxygen free radicals [8].

The HPA axis is a neuroendocrine system regulated by the circadian cycle [9, 10] and stress [11]. After being stimulated, neurons of the paraventricular nucleus of the hypothalamus release corticotropin-releasing hormone (CRH), which will be transported by hypothalamic-pituitary portal circulation and stimulate pituitary corticotroph cells to cleave proopiomelanocortin (POMC) in adrenocorticotrophic hormone (ACTH) [12]. ACTH is released into the bloodstream and acts on melanocortin receptor type 2 (MC2R), situated in the *zona fasciculata* of adrenals, inducing an increase in expression and/or activity of steroidogenic enzymes, including 11*β*-hydroxysteroid dehydrogenase-type 1 (11*β*-HSD1) and steroidogenic acute regulatory protein (StAR), and releasing glucocorticoids to the bloodstream [13]. The basal activity of the HPA axis is regulated by a negative feedback which is mediated through activation of glucocorticoid receptor (GR) and mineralocorticoid receptor (MR), located in the pituitary and hypothalamus, by glucocorticoids [14, 15].

The HPA axis is the main neuroendocrine system that regulates responses to stress. The production of high levels of ROS into the glands that comprise the HPA axis is associated with the activation of a stress-response system in several models of stress, including social isolation [16] and inflammatory and infectious diseases [17]. HPA axis hyperactivity induced by redox imbalance may occur by a reduction in negative feedback through a decrease in GR translocation to the cellular nucleus in corticotroph cells of the pituitary [17].

Although the free radical theory and the oxidative damage theory describe that accumulation of oxidative damage in cellular macromolecules is immensely toxic, ROS products by normal cell metabolism are vital to cell homeostasis maintenance, especially for its roles in immunocompetence and activation of several signal transduction pathways [18, 19]. Indeed, the antioxidant therapy presents several side effects, which is opposed to the anticipated properties of these substances [3]. Our hypothesis is that any imbalance in the redox system alters the homeostasis of the HPA axis culminating in its hyperactivation, and not just an increase in ROS production as shown in several diseases. Here, we undertook this study to evaluate the putative mechanism underlying the antioxidant-induced hyperactivation of the HPA axis in Wistar rats.

## 2. Materials and Methods

### 2.1. Animals and Treatments

Male Wistar rats (250–300 g) were obtained from the Oswaldo Cruz Foundation breeding colony and used in accordance with the guidelines of the Committee on Use of Laboratory Animals of the Oswaldo Cruz Foundation (CEUA-FIOCRUZ, license LW-23/11). Rats were housed in groups of three in a temperature-, humidity-, and light-controlled (12 h light : 12 h darkness cycle) colony room. Rats were given ad libitum access to food and water.

Twelve male rats were randomly assigned into 2 groups as follows: control (*n* = 6) and treated with the antioxidant N-acetyl L-cysteine (NAC) (*n* = 6). In another set of experiments, twelve male rats were randomly assigned into 2 groups as follows: control (*n* = 6) and treated with the antioxidant vitamin E (*n* = 6). In a third set of experiments, twenty male rats were randomly divided into 4 experimental groups: control (*n* = 5), treated with NAC (*n* = 5), treated with the exogenous glucocorticoid dexamethasone (*n* = 5), and treated with NAC (*n* = 5) plus dexamethasone. The rats were treated with NAC (150 mg/kg body weight) [20] or vitamin E (*α*-tocopherol, 40 mg/kg body weight) [21] by gavage once a day, during 18 consecutive days. Control rats received an equal volume of vehicle (sterile saline 0.9% and DMSO 0.1%, resp.). To analyze corticoid-induced negative feedback sensitivity, a group of animals received dexamethasone (0.02 mg/kg body weight, s.c.) daily, for 5 consecutive days [22], beginning 13 days after the starting of antioxidant treatment.

### 2.2. Corticosterone Quantification

Animals were euthanized in a CO_2_ chamber, during the nadir (08:00 h) of the circadian rhythm as described previously [23], and the blood was immediately collected from the abdominal aorta with heparinized (400 U/ml) saline. Plasma was obtained after sample centrifugation for 10 min at 1000 ×g and stored at −20°C until use. Plasma corticosterone levels were detected by radioimmunoassay (RIA) following manufacturer's guidelines (MP Biomedicals, Solon, OH, USA). Briefly, this is a competitive assay between the hormone presented in the sample and the hormone labelled with radioisotope (I^125^) to bind to a specific antibody. Thereby, an increase in amount of the hormone in the sample leads to a corresponding decrease in the fraction of labelled hormone bound to the antibody. Radioactivity quantification was carried out using a gamma counter (ICN Isomedic 4/600 HE; ICN Biomedicals Inc., Costa Mesa, CA, USA), and the amount of corticosterone in samples was calculated by interpolation to a standard curve performed in parallel.

### 2.3. Immunohistochemistry Staining

The adrenals and pituitary glands were immediately dissected after perfusion of rats with 0.9% sterile saline. Adrenals were quickly removed from the rats and cleaned of surrounding fat, while the pituitary glands were gently collected after the decapitation of rats and removal of the brain [24, 25]. Instantly after dissection, the glands were fixed in Milloning and embedded in paraffin. Paraffin-embedded sections of 3 *μ*m of rat pituitary and adrenals were deparaffinized with xylene, rehydrated by a graded series of ethanol washes, and boiled in sodium citrate buffer (10 mM, pH 6.0) at the temperature of 95°C for 15 min to enhance antigen retrieval. Tissue sections were incubated with 3% H_2_O_2_ in methanol for 20 min to block endogenous peroxidases. To prevent nonspecific binding, sections were then incubated for 3 h with a solution containing 2.5% bovine serum albumin (BSA), 8% fetal bovine serum (FBS), and 1% of nonfat milk dissolved in Tris-buffered saline enriched with 0.1% Tween 20 (TBST). After blocking, sections were incubated with primary specific antibody (polyclonal rabbit anti-rat StAR (1 : 50), GR (1 : 250), or MC2R (1 : 250) and polyclonal goat anti-rat MR (1 : 50), HO-1 (1 : 100), or Nrf2 (1 : 100) from Santa Cruz Biotechnology, Santa Cruz, CA, USA) diluted in TBST with 1% BSA overnight at 4°C.

Then, primary antibody binding was detected after incubating sections with a horseradish peroxidase conjugated-secondary antibody (polyclonal anti-goat or anti-rabbit IgG HRP, R&D System, Minneapolis, MN, USA) for 2.5 h, followed by a 20 min exposure to the HRP substrate 3-amino-9-ethylcarbazole (AEC). Sections were washed with TBST between all steps and weakly counterstained with hematoxylin for the easy identification of tissue structures. Finally, tissue sections were mounted in aqueous medium and images digitized via scanner microscope (Pannoramic SCAN150, 3D Histech, Budapest, Hungary) using a 20x objective lens. Images obtained from the anterior pituitary or *zona fasciculata* of the adrenal cortex were analyzed with Image Pro Plus 6.2 software (Media Cybernetics). Briefly, red to brown colored pixels associated with a positive immunohistochemistry stain were selected in a model image and applied to the remaining fields. The number of positive pixels was divided by the field area and expressed as pixels/*μ*m^2^.

### 2.4. Chemicals

Sodium citrate, AEC, NAC, vitamin E, dexamethasone, and hydrogen peroxide were purchased from Sigma Chemical Co. (Saint Louis, MO, USA); ethanol, methanol, and xylene from Merck (Rio de Janeiro, RJ, Brazil); and sodium heparin from Roche (São Paulo, SP, Brazil). All solutions were freshly prepared immediately before use.

### 2.5. Statistical Analysis

The data are reported as the mean ± standard error of the mean (SEM). All data were evaluated to ensure normal distribution. The assay of corticoid-induced negative feedback sensitivity was analyzed by one-way ANOVA followed by a Student-Newman-Keuls post hoc test, while all the other results were statistically analyzed by unpaired *t*-test, with Graphpad Prism 5.0. Probability values (*p*) of 0.05 or less were considered significant.

## 3. Results

### 3.1. Antioxidant Therapy Increases Plasma Corticosterone Levels in Wistar Rats

**Figure 1:**
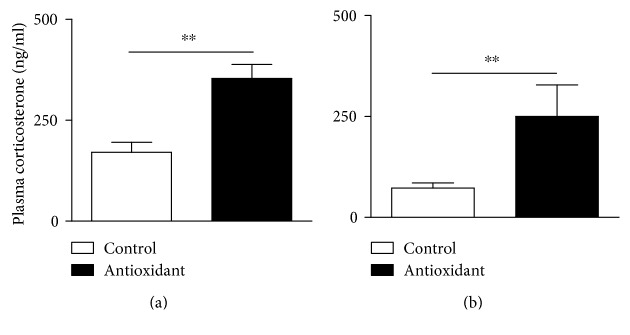
Antioxidant treatment increases circulating levels of plasma corticosterone in Wistar rats. (a) NAC (150 mg/kg, oral route) and (b) vitamin E (40 mg/kg, oral route) were given daily for 18 consecutive days. Untreated animals received an equal amount of vehicle (saline 0.9% or DMSO 0.1%). Data are expressed as the mean ± SEM of 6 animals. This result is a representative of two independent assays. ^∗∗^*p* < 0.01.

Initially, we investigated the impact of antioxidant therapy on circulating corticosterone levels. We observed that rats treated with either NAC (Figure 1(a)) or vitamin E (Figure 1(b)), for 18 consecutive days, presented a significant increase in plasma corticosterone levels compared to controls (mean ± SEM, *n* = 6; *p* < 0.01; two-tailed *t*-test).

### 3.2. NAC Induces Adrenal Hypertrophy and Upregulation of ACTH Receptor and StAR in the Adrenal Cortex of Wistar Rats

**Figure 2:**
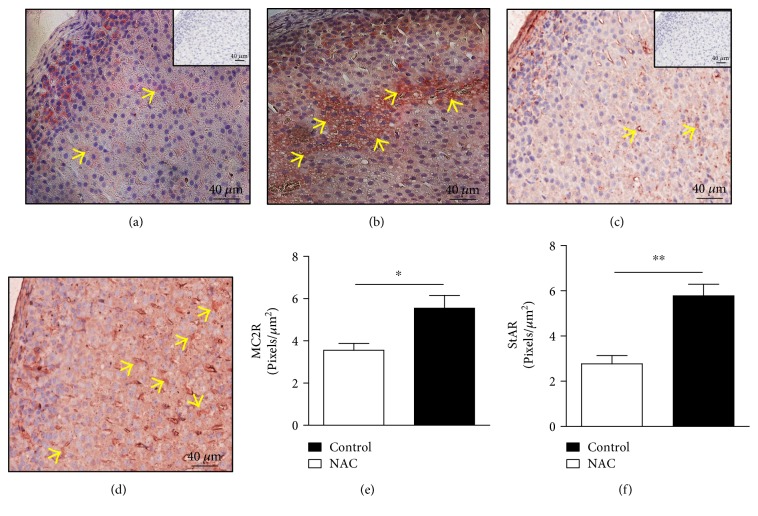
NAC induces an upregulation of MC2R and StAR expression in the *zona fasciculata* of the adrenal of Wistar rats. NAC (150 mg/kg, oral route) was given daily for 18 consecutive days and the analysis was made by immunohistochemistry. The panels show representative photomicrographs of adrenal expression of MC2R in the (a) control and (b) NAC-treated rats and StAR in (c) control and (d) NAC-treated rats. The quantification of pixels associated with MC2R and StAR expression is shown in (e) and (f), respectively. Inserts represent negative controls. Yellow arrows indicate immunolabelling of MC2R (a, b) and StAR (c, d) in the *zona fasciculata* of adrenals. Data are expressed as the mean ± SEM of 6 animals. This result is a representative of two independent assays. ^∗^*p* < 0.05 and ^∗∗^*p* < 0.01.

We hypothesized that the high corticosterone levels were due to increased stimulation of the adrenal cortex. We noted that NAC induced adrenal hypertrophy, as evidenced by the ratio between adrenal weight (mg) and body weight (g). The values of adrenal/body weight ratio increased from 0.066 ± 0.003 in control rats to 0.095 ± 0.008 (mean ± SEM, *n* = 6; *p* < 0.01; two-tailed *t*-test) in NAC-treated rats. The absolute adrenal weights were 24 ± 1.8 mg and 30 ± 1.5 mg (mean ± SEM, *n* = 6; *p* < 0.05; two-tailed *t*-test) to control and NAC-treated rats, respectively. In parallel, we showed that treatment with NAC increased the expression of ACTH receptor (MC2R) (Figures 2(b) and 2(e)) and steroidogenic enzyme StAR (Figures 2(d) and 2(f)) in the *zona fasciculata* of the adrenal cortex compared to that of control rats (Figures 2(a) and 2(c), resp.) (mean ± SEM, *n* = 6; *p* < 0.05 and *p* < 0.01, resp.; two-tailed *t*-test). The expression of MC2R is located in the membrane and cytoplasm of cells (Figures 2(a) and 2(b)), while StAR is expressed only in the cytoplasm of the cells (Figures 2(c) and 2(d)).

### 3.3. NAC Decreases Nrf2 and HO-1 Expression in the Anterior Pituitary of Wistar Rats

**Figure 3:**
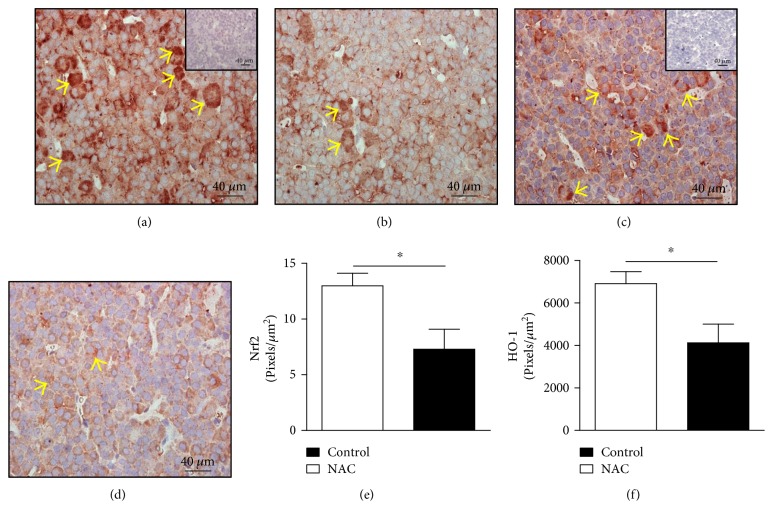
NAC reduces Nrf2 and HO-1 expression in the anterior pituitary of Wistar rats. NAC (150 mg/kg, oral route) was given daily for 18 consecutive days and analysis was made by immunohistochemistry. The panels show representative photomicrographs of pituitary expression of Nrf2 in (a) control and (b) NAC-treated rats and HO-1 in (c) control and (d) NAC-treated rats. The quantification of pixels associated with Nrf2 and HO-1 expression is shown in (e) and (f), respectively. Inserts represent negative controls. Yellow arrows indicate immunolabelling of Nrf2 (a, b) and HO-1 (c, d) in the anterior pituitary. Data are expressed as the mean ± SEM of 6 animals. This result is a representative of two independent assays. ^∗^*p* < 0.05.

Our next approach was to determine if the treatment with NAC could interfere with the expression of antioxidant arsenal in the anterior pituitary, an important component of the HPA axis which regulates corticosterone production by adrenals. Treatment with NAC reduces the expression of transcription factor Nrf2 (Figures 3(b) and 3(e)) and the antioxidant enzyme HO-1 (Figures 3(d) and 3(f)) in the anterior pituitary compared to that of control rats (Figures 3(a) and 3(c), resp.) (mean ± SEM, *n* = 6; *p* < 0.05 and *p* < 0.05, resp.; two-tailed *t*-test). The expression of Nrf2 is located in the nucleus and cytoplasm of cells (Figures 3(a) and 3(b)), while HO-1 is expressed only in the cytoplasm of the cells (Figures 3(c) and 3(d)).

### 3.4. NAC Reduces Glucocorticoid Receptor Expression in the Anterior Pituitary and Impaired Negative Feedback of the HPA Axis in Wistar Rats

**Figure 4:**
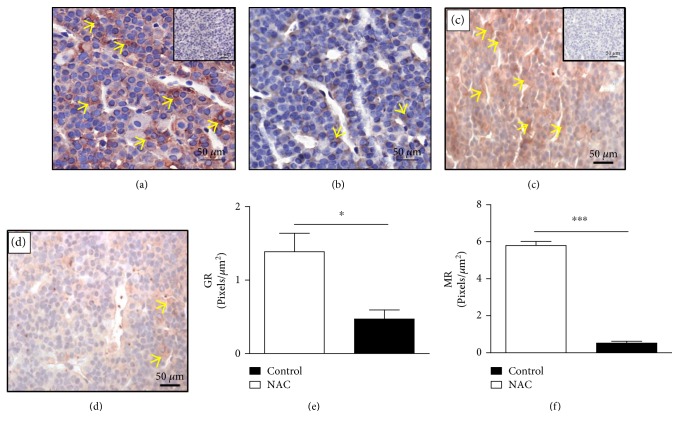
NAC decreases GR and MR expression in the anterior pituitary of Wistar rats. NAC (150 mg/kg, oral route) was given daily for 18 consecutive days and analysis was made by immunohistochemistry. The panels show representative photomicrographs of pituitary expression of GR in (a) control and (b) NAC-treated rats and MR in (c) control and (d) NAC-treated rats. The quantification of pixels associated with GR and MR expression is shown in (e) and (f), respectively. Inserts represent negative controls. Yellow arrows indicate immunolabelling of GR (a, b) and MR (c, d) in the anterior pituitary. Data are expressed as the mean ± SEM of 6 animals. This result is a representative of two independent assays. ^∗^*p* < 0.05 and ^∗∗∗^*p* < 0.001.

High levels of plasma corticosterone induced by antioxidants can also be associated with a failure in the negative feedback of the HPA axis. Treatment with NAC decreases the expression of both glucocorticoid receptors GR (Figures 4(b) and 4(e)) and MR (Figures 4(d) and 4(f)) in the anterior pituitary compared to that of control rats (Figures 4(a) and 4(c), resp.) (mean ± SEM, *n* = 6; *p* < 0.05 and *p* < 0.001, resp.; two-tailed *t*-test). The expressions of GR (Figures 4(a) and 4(b)) and MR (Figures 4(c) and 4(d)) are located in the nucleus and cytoplasm of cells.

**Figure 5:**
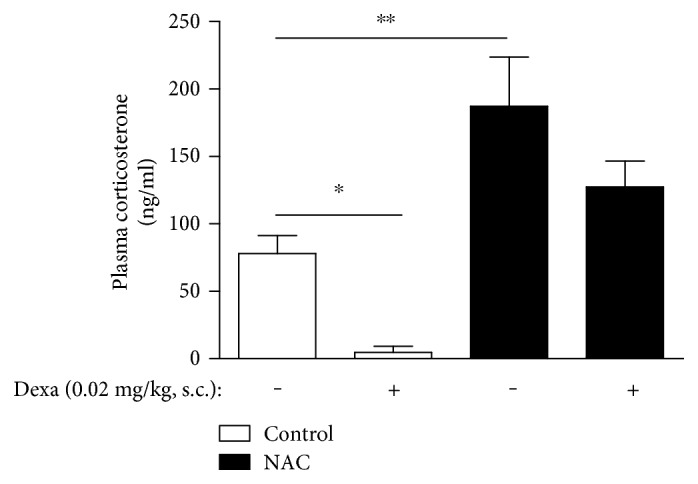
NAC impairs dexamethasone-induced negative feedback of the HPA axis in Wistar rats. NAC (150 mg/kg, oral route) was given daily for 18 consecutive days. Some groups of animals were injected with dexamethasone (0.02 mg/kg, s.c.) starting 13 days after the beginning of NAC treatment, daily during five consecutive days. Data are expressed as the mean ± SEM of 5 animals. This result is a representative of two independent assays. ^∗^*p* < 0.05 and ^∗∗^*p* < 0.01.

Thus, we treated rats with a low dose of dexamethasone (0.02 mg/kg, s.c.) and analyzed the circulating levels of corticosterone. We showed that dexamethasone induced a strong negative feedback response and reduced the plasma corticosterone levels in control rats (mean ± SEM, *n* = 6; *p* < 0.05; one-way ANOVA followed by a Student-Newman-Keuls post hoc test *t*-test); however, although treatment with NAC induced an increase in plasma corticosterone levels compared to controls (mean ± SEM, *n* = 6; *p* < 0.01; one-way ANOVA followed by a Student-Newman-Keuls post hoc test), dexamethasone did not alter the levels of corticosterone in NAC-treated rats (Figure 5()).

## 4. Discussion

This study investigated the role of antioxidants on the modulation of endogenous glucocorticoid levels. We found that treatment with antioxidants either NAC or vitamin E increases the plasma levels of corticosterone in rats, in association with an overexpression of ACTH receptor and the steroidogenic enzyme StAR in the adrenal glands. NAC also induces a drop in HO-1 and Nrf2 expression in the pituitary and blocked the ability of dexamethasone to perform negative feedback of the HPA axis by decreasing the expression of glucocorticoid receptors in the pituitary. Our findings suggest that antioxidants cause a hyperactivation of the HPA axis with a clear dependency of upregulation of ACTH receptor in adrenals and downregulation of glucocorticoid receptors in the pituitary.

In this study, we showed that both NAC and vitamin E increase circulating levels of corticosterone in rats. NAC and vitamin E are antioxidants that act through distinct mechanisms of action. While NAC provides cysteine, which is a precursor for reduced glutathione production, and scavenges oxidants directly, including hydroxyl radical, ^−^OH, and hypochlorous acid [8]; vitamin E is a peroxyl radical scavenger and due to its lipid solubility plays an important role in maintaining integrity of long-chain polyunsaturated fatty acids in the membranes of cells [6, 7]. The fact that prolonged treatment with two antioxidants with different mechanisms of action can increase circulating levels of corticosterone indicates that this is not an epiphenomenon, but suggests that inhibition of physiological levels of ROS in the HPA axis is responsible for its hyperactivity.

The HPA axis is the main neuroendocrine system that regulates responses to stress [8]. It is well known that the production of high levels of ROS into the glands that comprise the HPA axis is associated with the activation of a stress-response system [13, 14]. Therewith, antioxidant treatment reduces corticosterone levels in several models of diseases, including brain oxidative stress induced by lipopolysaccharide [26]. However, although accumulation of oxidative damage in cellular macromolecules is immensely toxic, ROS products by normal cell metabolism are vital to cell homeostasis maintenance [15, 16]. Our hypothesis is that physiological levels of ROS have a fundamental role in maintaining the homeostasis of the HPA axis. In fact, treatment with NAC in normal rats effectively reduced ROS levels in chondrocytes and Lin^−^CD45^+^AnV^−^ marrow cells [27], suggesting that in our model, the antioxidant therapy probably reduces intracellular ROS content in the adrenal and pituitary glands. Thereby, we strongly suggest that any imbalance in the redox system in glands which comprise the HPA axis culminates in its hyperactivation.

In an attempt to elucidate how antioxidants induce the production of glucocorticoids by the HPA axis, we analyzed the expression of adrenal MC2R. Prolonged treatment with NAC increases adrenal MC2R expression. This higher adrenal MC2R expression after treatment can explain, at least partially, the capacity of NAC to increase circulating glucocorticoid levels. Increased expression of MC2R may lead to high activation of this receptor by ACTH and induction of the transcription of several key genes of enzymes involved in steroidogenesis, including StAR [28]. In fact, we showed that NAC induces an upregulation in the expression of StAR into the adrenal glands. StAR rapidly transports cholesterol to the inner mitochondrial membrane, where the conversion of this steroid precursor into pregnenolone, a precursor of steroid hormones, occurs [29]. This metabolic step is crucial to rapid glucocorticoid production into the adrenals in a stress stimulus, once steroidogenic cells store very little amount of glucocorticoids [10].

Although the increased expression of MC2R and StAR alone may explain the increase in glucocorticoids levels, other molecular alterations can also participate in the HPA axis hyperactivity noted after antioxidant treatment. Once the HPA axis is finely regulated by a negative feedback response on the hypothalamus and/or pituitary that normalizes circulating corticosterone levels, we hypothesized that antioxidants could induce a defect in the negative feedback regulation in the HPA axis. We observed that treatment with NAC downregulated the expression of Nrf2 in the anterior pituitary gland of rats. Nrf2 is a transcription factor that regulates expression of several antioxidant enzymes, including superoxide dismutase, catalase, glutathione peroxidase, and HO-1 [30]. Although NAC can induce upregulation of Nrf2 expression in phosgene-induced acute lung injury [31], our data is in accordance with others which described that NAC inhibited Nrf2 expression in lymphoid malignant cell lines stimulated with honokiol [32], suggesting that the effect of antioxidant NAC on Nrf2 expression depends on the cell type and condition of the study. Furthermore, we noted that NAC also decreased the expression of HO-1 in the anterior pituitary of rats. The drop in HO-1 levels after treatment with NAC indicates that the low content of Nrf2 is associated with a reduced ability of this transcription factor to induce production of antioxidant enzymes by pituitary cells. We suppose that the downregulation of Nrf2 expression is a strategy of the organism to maintain homeostasis in rats treated for several days with NAC. In fact, it has been shown that exogenous antioxidants can reduce the expression and/or activity of endogenous antioxidant enzymes [33, 34]. These data indicate that the pituitary as well as adrenals is also a direct target of antioxidant drug effects.

Our next approach was to investigate the sensitivity of the HPA axis to negative feedback induced by synthetic glucocorticoid in NAC-treated rats. Dexamethasone decreased plasma corticosterone levels in control rats; however, it did not alter circulating glucocorticoid amount in rats treated with NAC, showing that antioxidants abolish the ability of glucocorticoids to perform negative feedback of the HPA axis. NAC treatment also decreased expression of both GR and MR in the pituitary of rats, indicating that a reduction in glucocorticoid receptor expression in the pituitary of rats can explain the inability of dexamethasone to induce negative feedback of the HPA axis in NAC-treated animals. Our data confirmed the capacity of NAC in inhibiting GR expression, once NAC decreases GR protein levels in the hypothalamus of mice fed with a high-cholesterol diet [35].

Currently, many people consume dietary supplementation with antioxidants to combat diseases associated with aging [36]; however, several clinical trials testing benefits and harms of antioxidant supplements found that antioxidants have been unable to demonstrate beneficial effects and pointed that they seemed to cause an increase in all-cause mortality [37–40]. Once antioxidants induce a HPA axis dysfunction with concomitant increased levels of circulating glucocorticoids, this food supplement is shown as a risk to human health. This occurs because the hyperactivation of the HPA axis, and consequently the glucocorticoid signaling system, may alter the epigenetic landscape and influence genomic regulation and function conducting to the development of aging-related diseases [41]. Some harmful effects of hypercorticoidism, which can culminate with aging-related diseases, are deleterious effects on the central nervous system (CNS), including neuroinflammatory environment, loss of neuronal function, and apoptosis of neuronal cells, causing a decrease in hippocampal neurogenesis and an increase in neuroinflammation and neurodegeneration [42–44]. These deleterious effects of hypercorticoidism on the CNS can lead to the development of a variety of progressive neurodegenerative and psychiatric diseases, including schizophrenia, dementia, depression, Huntington's disease, and Alzheimer's disease [45–49]. Although we showed that antioxidants can induce high production of glucocorticoids, it is well known that chronic stress promotes redox imbalance throughout the body [50], as in blood of humans [51–53], such as in several structures of the CNS of rats including the frontal cortex, hypothalamus, and hippocampus [54]. These data suggest that chronic stress accelerates cellular aging through inducing increased levels of ROS [50]. These observations are conflicting with ours, once we show that antioxidants decreased the expression of Nrf2 and HO-1. However, the induction of ROS production is not the only mechanism related to stress-induced cellular aging. Chronic stress reduces brain-derived neurotrophic factor (BDNF) in the hippocampus and prefrontal cortex and increases neuroinflammation, an alteration noted in the formation of depression [44]. Furthermore, chronic stress induces prolonged periods of glutamate release in the hippocampus and decreases the ability to clear extracellular glutamate. These alterations in the glutamate transmission may be related to the impairments in the spatial and contextual memory performance and stress-associated psychiatric disorders, including mood and anxiety [55].

In addition, hypercorticoidism can also increase susceptibility to cancer [56], one of the most important aging-related diseases. Although ROS can cause oncogenic mutations and activate oncogenic pathways [57], dietary supplementation with antioxidants promotes increased incidence and death from lung and prostate cancer [58]. Furthermore, antioxidants induce melanoma progression by promoting metastasis [59]. One possibility is that the high incidence of cancer in people which use dietary supplementation with antioxidants can be related to hyperactivation of the HPA axis.

In summary, our results indicate that antioxidant therapy can induce an activation of the HPA axis, with an increase in the levels of systemic glucocorticoids by upregulating ACTH receptor in the adrenal and downregulating glucocorticoid receptors in the pituitary. Thereby, indiscriminate use of antioxidant supplements can be a risk to develop several morbidities related to persistent hypercorticoidism, as observed in Cushing's disease.
